# Efficacy and Predictive Factors of Oral Spironolactone Treatment in Chronic Central Serous Chorioretinopathy

**DOI:** 10.1155/2024/7197249

**Published:** 2024-03-18

**Authors:** Sheng Gao, Yun Zhang, Meixia Zhang

**Affiliations:** ^1^Department of Ophthalmology, West China Hospital, Sichuan University, Chengdu, China; ^2^Research Laboratory of Macular Disease, West China Hospital, Sichuan University, Chengdu, China

## Abstract

**Purpose:**

To evaluate the efficacy of spironolactone in the treatment of chronic central serous chorioretinopathy (CSC) and identify imaging characteristics that can predict the benefit of spironolactone treatment.

**Methods:**

Patients with chronic CSC were treated with spironolactone (20 mg/tid) and followed for 6 months. The primary outcome measure was complete resolution of the subretinal fluid (SRF), and the best-corrected visual acuity (BCVA), the SRF area, the central macular thickness (CMT), the subfoveal choroidal thickness (SFCT), and the density of the choriocapillaris vessel and adverse events were secondary outcome measures. Patients who presented complete resolution of SRF were included in the responder group and the other patients who had moderate or no resolution were included in the nonresponder group. Imaging characteristic comparisons between the responder and nonresponder groups were performed with regression analyses to identify factors that are predictive of a good response to treatment.

**Results:**

Forty-two eyes of 42 patients with a mean age of 46.06 ± 6.66 years were included. A total of 57.1% of the patients achieved a complete resolution of SRF. The mean SRF area, CMT, and SFCT decreased significantly (all *P* < 0.05) throughout the follow-up period and BCVA improved slightly (*P* > 0.05). The vascular density of the choriocapillaris of the fellow eyes did not vary significantly during treatment. Logistic regression analysis revealed that SFCT (*P*=0.002) and the intact ellipsoid zone (*P*=0.001) were correlated with disease resolution. A relatively higher baseline SFCT was a predictive factor associated with a good response to treatment according to multivariate analysis.

**Conclusions:**

This study suggested that oral spironolactone could be an effective and safe therapy for chronic CSC patients. Eyes with a higher baseline SFCT and intact ellipsoid zone could have a good response. These parameters are an important prognostic marker.

## 1. Introduction

Central serous chorioretinopathy (CSC) is characterized by focal serous retinal detachment with or without pigment epithelium detachment and is typically observed in working-age men [1, 2]. Currently, it is generally believed that the primary lesion of CSC is located in the choroid and belongs to the pachychoroid spectrum diseases. These CSC patients with pachychoroid are usually prone to relapse, causing atrophic retinal pigment epithelium (RPE) and RPE dysfunction. Increasing evidence has supported that the corticosteroids acts as an important risk factor for CSC [1]. Exogenous glucocorticoids or endogenous cortisol metabolism disturbances trigger or even aggravate serous retinal pigment epithelial detachments [3]. In fact, primary adrenal cortical steroid hormones act by binding to structurally similar mineralocorticoid receptors (MRs). Competitive MR antagonists are likely to block the binding of corticosteroids to this receptor [4]. Spironolactone, the first competitive steroidal MR antagonist, is used mainly to treat hypertension, hyperaldosteronism, and congestive heart failure [5]. An animal model of CSC showed a good response to treatment with MR antagonists [6, 7], and several studies have reported that spironolactone administered orally at a low dose seems to have a good effect on chronic or nonself-limiting CSC, with retinal and choroidal anatomical recovery and potential visual acuity improvement [8]. The noninvasive and well-tolerated oral administration of spironolactone is also an advantage. Based on multimodal imaging, chronic CSC patients with potential imaging predictors might benefit from spironolactone treatment. Identifying these possible prognostic markers will help us differentiate responders who would benefit from MR antagonists, which is the lacuna of published studies.

Therefore, the purpose of this study was to evaluate the efficacy of spironolactone in eyes with chronic CSC and to identify specific prognostic markers that can distinguish responders who would benefit immediately from MR antagonist treatment.

## 2. Methods

### 2.1. Study Design and Follow-Up Examinations

This was an interventional retrospective clinical trial. Forty-five eyes of 45 patients (30 men and 15 women) with chronic CSC who underwent spironolactone treatment at the West China Hospital of Sichuan University between December 2021 and December 2022 were retrospectively studied. The study adhered to the principles of the Declaration of Helsinki and was approved by the Ethics Committee of the Sichuan University Biomedical Research West China Hospital.

Chronic CSC was defined as persistent subretinal fluid (SRF) associated with changes in RPE or leakage on fundus fluorescein angiography (FFA) accompanied by visual symptoms for at least 4 months. The inclusion criteria included chronic CSC patients who received spironolactone treatment, patients 18 years or older, and patients with no spontaneous resolution. Exclusion criteria included any other macular diseases; ocular surgery, previous photodynamic therapy (PDT), intravitreal injections of antivascular endothelial growth factor (anti-VEGF), or retinal laser photocoagulation within one year prior; a follow-up period of less than 6 months; and systemic contraindications to spironolactone, such as severe kidney or liver disease.

All patients underwent a complete history, including age, sex, first episode or recurrence of CSC, and duration of onset.The ophthalmological tests included BCVA, dilated fundus biomicroscopy; EDI-OCT (Heidelberg Engineering); and OCTA (Carl Zeiss Meditec). FFA and indocyanine green angiography (ICGA) (Heidelberg) were performed at baseline to exclude other macular diseases. Serum electrolytes, blood pressure, and liver and kidney functions were recorded during the follow-up period.

All included patients were treated with spironolactone (Jiangsu Zhengda Fenghai Pharmaceutical Co., Ltd.) at a dose of 20 mg three times a day for 3 months. The patients were informed about the off-label use of the drug and signed an informed consent prior to treatment. The drug was discontinued if systemic adverse events occurred, including hyperkalemia (>5 mmol/L), hypotension (<90/60 mmHg), elevated serum creatinine (>106 *µ*mol/L), gastrointestinal upset, or hypersensitivity reactions. In cases where signs and symptoms resolved completely in advance, treatment was stopped early after a two-week consolidation period. Patients who demonstrated *a* > 50% reduction but no total regression had therapy extended to 6 months.

Patient records were reviewed at follow-up visits at the beginning of the study and at the first, third, and sixth months after spironolactone treatment. At follow-up visits, BCVA, slit lamp examination, EDI-OCT, OCTA, blood pressure, and serum electrolytes were examined and recorded.

### 2.2. Multimodal Imaging

EDI-OCT was performed with twelve radial scans centered on the fovea. The subfoveal choroidal thickness (SFCT) was measured from the outer edge of the RPE to the inner border of the sclera using features of the instrument software. Central macular thickness (CMT) was measured using a macular thickness map with the software. The SRF area was sketched with the contours of the highest neurosensory detachment on the b-scan images and assessed in square millimeters by automeasurement with software. To obtain the most accurate data on the change in SRF, this manual measurement was performed at the highest point of neurosensory detachment and at each follow-up visit. All EDI-OCT findings and measurements were performed independently by two examiners (SG and YZ). If the measurements differed by more than 10%, a third examiner performed the measurements. The pathological findings of EDI-OCT were evaluated in the area of the 1500 *µ*m radius centered by the fovea, including pigment epithelium detachments (PEDs), double layer signs, the integrity of the ellipsoid zone (whether the ellipsoid zone is intact), the integrity of the external limiting membrane (ELM) (whether the ELM is intact), hyperreflective foci in the outer segment (OS) and outer nuclear layer (ON layer), and subretinal deposits in the subretinal fluid. The ellipsoid zone and the ELM layer were considered intact if there was no interruption in that layer on any of the OCT scans in the area of the radius of 1500 *µ*m radius centered by the fovea.

OCTA was performed using a Zeiss prototype (Cirrus HD-OCT 5000, Carl Zeiss Meditec), which is based on the optical microangiography algorithm [9]. OCTA software offers the option of 6 mm × 6 mm OCT angiograms and automated segmentation of full-thickness retinal scans. AngioPlex incorporates FastTrac retinal tracking technology to reduce motion artifacts. All scans were independently completed by a single examiner (YZ) for quality evaluation. Substandard scans in which the signal strength (SS) fell below the manufacturer's recommended cut-off point (SS < 7) and images with significant eye movement were excluded. Choriocapillaris density (CCD) was measured by OCTA, and Sattler + Haller vessel density was quantified by en face images to avoid sensitivity roll-off with increasing imaging depth [10, 11]. To avoid the shadowing effect of neurosensory detachment that affects the OCTA flow signal in the choriocapillaris, we applied the CCD of a fellow eye for analysis [12]. Previously developed and validated custom MATLAB software (MathWorks, Inc., Natick, MA, USA) was used to process and analyze these OCTA images or en face images [13, 14]. In brief, the density algorithm included three key steps: constructing the binary location of large retinal vessel shadows; capillary segmentation using the modified Otsu algorithm; and calculating the density values of the choroidal vasculature. For eyes with more than one hyperfluorescent area on the FFA and ICGA, the data from the largest area were used for statistical analysis.

### 2.3. Statistical Analysis

The variables included in our analysis were age, sex, the proportion of subretinal fluid resolution, BCVA, SRF area, CMT, SFCT, CCD, and Sattler + Haller vessel density. The aim of CSC treatment is to preserve the outer layers of the neurosensory retina and achieve complete resolution of SRF. Therefore, the complete elimination of SRF to restore normal anatomical and functional photoreceptor-RPE interactions should be the main surrogate endpoint in intervention trials for chronic CSC. The patients were divided into 2 study groups based on their responses to SRF resolution. The responder group was considered to have complete resolution of the subretinal fluid. The nonresponder group was considered to be patients with moderate or no resolution of the subretinal fluid. Snellen BCVA was converted into the logarithm of the minimal angle of resolution (logMAR) score for statistical analysis [15]. Continuous data are presented as mean ± standard deviation (SD) and categorical variables as counts and percentages. The Kolmogorov–Smirnov test was used to assess the distribution patterns of the data. Comparisons between variables were performed using the Wilcoxon paired signed-rank test, independent samples Student's *t*-test, Wilcoxon signed-rank sum test, and Fisher's exact test. Repeated measures analysis of variance was used for statistical comparisons of repeated measurements. Univariate regression analysis was performed to identify factors that predict visual outcomes. Multivariate binomial logistic regression analysis was performed to analyze predictors of disease resolution. The SRF' complete resolution curves in both the responder and nonresponder groups were drawn by using the Kaplan–Meier curve (GraphPad Prism software). All data were evaluated using SPSS Version 25.0 (IBM, Armonk, New York, USA). A *p* value < 0.05 was considered to indicate statistical significance.

## 3. Results

### 3.1. Patient Characteristics

Forty-two eyes of 42 patients (29 men and 13 women) were included in the study. Three patients were excluded from the final analysis; these included two individuals who were lost to follow-up and one who requested conversion to other therapies. The mean ± SD age of the patients was 46.06 ± 6.66 years. The mean ± SD disease duration before treatment was 7.16 ± 2.97 months.

### 3.2. Treatment Effect

**Figure 1:**
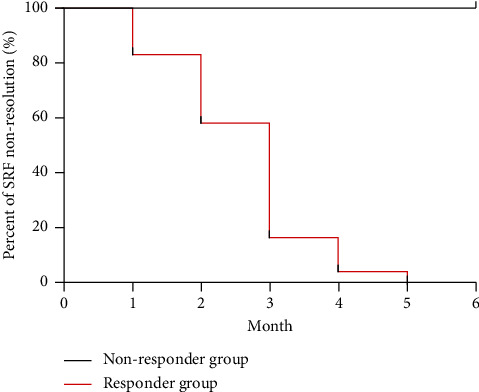
The percent of SRF nonresolution patients in the nonresponder and responder groups during the 6-month follow-up period. In the responder group (24 eyes), foveal subretinal detachment achieved complete resolution in 4 eyes (16.7%) at 1 month, in 10 eyes (41.7%) at 2 months, in 20 eyes (83.3%) at 3 months, in 23 eyes (95.8%) at 4 months, and in 24 eyes (100%) since the fifth month.

A complete resolution of subretinal fluid in responder patients was observed in 24 of 42 eyes (57.1%) after 6 months of treatment; these patients comprised the responder group. In the responder group, the SRF achieved a complete resolution curve as shown in Figure 1, in which most eyes (20 eyes, 83.3%) achieved complete resolution at 3 months. Treatment was stopped early in 4 eyes due to complete resolution in advance. Among patients who stopped treatment forward, SRF recurrence was detected in 3 eyes 2 months after discontinuation of treatment. No obvious treatment effect (incomplete resolution of SRF) was observed in 18 eyes (42.9%), which corresponded to the nonresponder group. Visual acuity efficacy and imaging data of all patients at 1, 3, and 6 months are summarized in Table 1. There was a certain increase in visual acuity and OCT imaging, which revealed that the mean SRF area, as well as the mean CMT and the mean SFCT, decreased significantly during follow-up (*P* < 0.05). OCTA imaging revealed that the choriocapillaris density of fellow eyes and the Sattler + Haller density did not change significantly after spironolactone treatment.

### 3.3. Comparison of Treatment Efficacy between Responders and Nonresponders

Visual acuity and imaging data of the two groups are shown in Table 2. The patients in the two groups had an increased visual acuity at 6 months compared to baseline, while the mean change in visual acuity was not significantly greater in the responder group than in the nonresponder group (*P*=0.49). However, full recovery from visual acuity to 20/20 Snellen BCVA was observed more frequently in the responder group (11 patients versus 4 patients).

The SRF area in the responder group decreased to complete resolution at 6 months compared to that at baseline, while the nonresponder group showed a decrease in the mean SRF area to 0.15 ± 0.10 mm^2^. The mean change in the SRF area was significantly greater in the responder group than in the nonresponder group at each follow-up time and was similar to the mean change in CMT, which also decreased significantly in the responder group. In particular, the mean change in SFCT in the entire cohort was significantly lower, but there were no significant differences between the responder and nonresponder groups, except for the baseline SFCT (497.50 ± 99.36 vs 365.50 ± 119.04, *P*=0.002).

### 3.4. Comparison of Clinical Characteristics between Responders and Nonresponders

The demographic data and baseline characteristics comparisons between the two groups are summarized in Table 3. No significant difference was observed between the two groups for clinical characteristics at the baseline visit. However, it is worth noting that the baseline visual acuity in the responder group was slightly better than that in the nonresponder group, albeit not significantly.

The baseline SFCT and ellipsoid integrity were found to be significant factors for distinguishing the response to spironolactone treatment. In patients who showed a complete regression of subretinal fluid at 6 months of follow-up, the frequency of thicker SFCT and a continuous ellipsoid zone was significantly greater at baseline. There was no statistically significant difference in frequency between the responder and nonresponder groups in terms of the frequency of PED, double layer sign, ELM integrity, hyperreflective foci in the OS and ON layers, subretinal deposits, baseline vessel density of the choriocapillaris and Sattler + Haller layers, or abnormal choroidal vessels of the choriocapillaris on OCTA.

**Figure 2:**
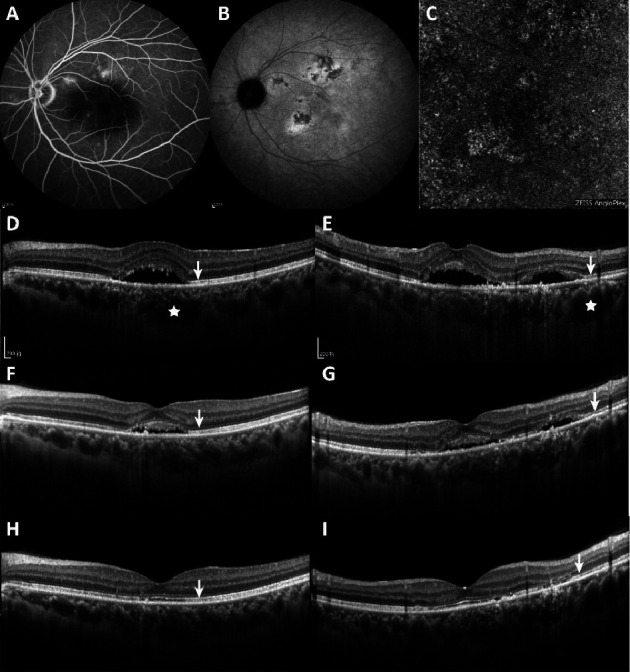
Multimodal images of a 44-year-old man with chronic central serous chorioretinopathy for 4 months. (A) Fluorescein angiography image showing slight leak points. (B) Indocyanine green angiography image showing several areas of hyperfluorescence and hypofluorescence indicating choroidal hyperpermeability. (C) Optical coherence tomography angiography image (6 × 6 mm) of the choriocapillaris plexus. (D) Horizontal- and (E) vertical-enhanced depth optical coherence tomography images of a responder to spironolactone at baseline, centered on the fovea, showing foveal subretinal detachment accompanied by increased choroidal thickness at baseline (white stars) and an intact ellipsoid zone (white arrow). After spironolactone treatment, a gradual resolution of the subretinal fluid was observed after 1 month (F, G), and complete resolution of the subretinal detachment was observed after 3 months of spironolactone treatment, lasting up to 6 months (H, I).

Finally, multivariate binomial logistic regression analysis was performed to identify the most significant predictor of good anatomical response to spironolactone treatment. According to the final model, a relatively higher baseline SFCT was a significant and independent predictor of the complete resolution of SRF (odds ratio: 0.987, 95% CI: 0.977–0.998; *P*=0.016). Moreover, there was no significant difference in ellipsoid integrity, which is a crucial predictor of the complete resolution of SRF (odds ratio: 0.192, 95% CI: 0.03–1.22; *P*=0.08) (Figure 2). However, ellipsoid integrity was positively correlated with the resolution of the subretinal fluid. In the responder group, 19 of 24 eyes (79.1%) had an intact ellipsoid zone, while 3 of 18 eyes (16.7%) had an intact ellipsoid zone (*P*=0.001).

### 3.5. Safety Analysis

No patients experienced serious adverse events associated with treatment. A patient had a potassium level of 5.47 mmol/L at the 4-month of follow-up, which normalized after withdrawal from the drug for 2 weeks without other treatment. Furthermore, there was no statistically significant difference in the overall change in potassium levels (*P*=0.283). No patient reported gastric pain or dizziness. Blood pressure remained within the normal range (*P*=0.432). In general, the individuals tolerated spironolactone well.

## 4. Discussion

Persistent SRF associated with chronic CSC can be complicated by permanent loss of the outer segments of the photoreceptors, diffuse RPE atrophy, cystoid macular degeneration, and secondary CNV, which are considered risk factors for deterioration of visual acuity [16]. Thus, when self-regression is not expected, timely intervention is critical for long-term visual outcomes. In addition to the observation and exclusion of risk factors, current therapies for CSC include laser photocoagulation, PDT, subthreshold micropulse lasers, and several oral medications [4]. However, there is no consensus on the management of nonresolving or chronic CSC [17–20].

Spironolactone is the first competitive steroidal MR antagonist that has a chemical structure similar to that of aldosterone and has competitive MR-blocking capabilities [21]. The occurrence and development of CSCs involve overactivation or inappropriate activation of the MR pathway [4]. Zhao et al. demonstrated that intraocular injection of high-dose corticosterone or aldosterone can induce acute activation of the MR pathway in rat eyes. These patients exhibited choroidal vasodilation and leakage, lengthening of the microvilli of RPE cells, increased choroidal thickness, and accumulation of SRF. All of these findings are similar to those of the CSC phenotype. These results suggest that MR pathway blockade can be used to reverse choroid vasculopathy [6, 7]. Thus, oral MR antagonists may be an option for patients who are not suitable (such as those unable to identify leakage points or extensive SRFs) or who reject invasive treatment (such as PDT or lasers). Importantly, the MR antagonist was well-tolerated by patients, with minimal occurrence of adverse events [22]. Furthermore, oral administration may be effective in promoting the recovery of choriocapillaris in the affected and unaffected eyes of patients with chronic CSC [23, 24].

Our findings suggest that spironolactone has good effects on SRF, CMT, and SFCT. Approximately 57.1% of the patients achieved a complete resolution of the subretinal fluid after 6 months of follow-up treatment. Similar to our study, Bousquet et al. reported a pilot study in which the SRF was more highly resolved [25]. The follow-up period lasted 6 months in our study. Clinically, the high rate of recurrence observed in patients with chronic CSC indicates that prolonged treatment is necessary to ensure a complete resolution of SRF accumulation [26]. Furthermore, the diffuse damage degree of RPE photoreceptor cells at baseline is perceived to indicate the prognosis of visual acuity. In our study, after 6 months of treatment, patients with poor baseline vision had different degrees of SRF regression, but the BCVA did not improve in some patients due to diffuse damage to photoreceptor cells and atrophy of the outer retinal structure. Similarly, the VICI trial concluded that eplerenone was not superior to placebo in improving BCVA in patients with chronic CSC [27]. However, this study raised a few questions, including about the target sample size, the choice of the primary endpoint, good baseline vision, and several biomarkers previously reported to be associated with the response to eplerenone [28, 29]. It seems that the responders had a good visual acuity and less fluid at baseline than the nonresponders. However, there was no significant difference. These differences cannot be attributed to drug efficacy and must be tested in an RCT because CSC can resolve spontaneously over time, especially in patients with mild disease. This cannot be resolved in a retrospective, noncomparative trial. However, spironolactone might not always improve functional outcomes; rather, it appears to improve anatomical results and may have preventive effects in patients with long durations of chronic disease. Therefore, identifying potential imaging predictors that can predict a good response to spironolactone treatment is valuable.

Our study demonstrated that several potential imaging predictors can be used as tools to identify patients who might benefit mainly from spironolactone treatment. A thick choroid at the beginning of the study was a factor associated with the response to treatment according to the multivariate analysis. Our study suggested that spironolactone treatment was more effective in patients with a much thicker baseline choroid and could be considered a crucial and independent predictor of patients who would benefit from prompt MR antagonist treatment. This result is in line with that of a previous study [30, 31]. Singh et al. identified the factors that predict visual and anatomical outcomes in eyes with CSC and reported that OCT parameters such as changes in both CMT and SFCT could be predictive of disease resolution, while changes in CMT and baseline SRF height correlated well with changes in BCVA [32]. Treatment with spironolactone can reverse glucocorticoid-induced choroidal vasodilation and leakage. Patients characterized by a thick baseline SFCT, in which the subretinal fluid is probably due to enlarged choroidal vessels and increased choroidal hyperpermeability, thus responding almost constantly and completely to spironolactone treatment, promptly showed complete resolution of the subretinal fluid. SRF resolution accompanied by rapid choroidal thinning can reflect the efficacy of spironolactone in patients with a thick baseline choroid. In addition, the integrity of the ellipsoid zone at baseline was found to be positively correlated with a good anatomical response to spironolactone treatment. The integrity of the ellipsoid zone has been assumed to be highly clinically important in the diagnostic and prognostic evaluation of various retinal disorders [33]. We speculated that the reason for the correlation between the integrity of the ellipsoid zone and a good response to spironolactone was the presence of subretinal fluid. The discontinuous ellipsoid zone causes the accumulation of photoreceptor outer segment shedding, lipid- or protein-like compounds in the subretinal fluid, and changes in osmotic pressure, which are adverse to fluid resolution. Pump function insufficiency and damage to the RPE itself could also explain the lack of response in patients with a discontinuous ellipsoid zone.

OCTA evaluation of vessel density in CSC eyes has provided new insight into disease mechanisms. OCTA demonstrated hypoperfusion areas surrounded by hyperperfusion regions in the CC of CSC patients, which corresponded to late staining on ICGA [34]. However, the study of choroidal vessels in chronic CSC patients has been controversial. Toto et al. reported that the choroidal vascularity index was reduced in chronic CSC patients after oral eplerenone treatment in both the affected and fellow eyes during follow-up [24]. In our study, we only applied the choriocapillaris vascular density of fellow eyes from OCTA images to avoid the shadowing effect of neurosensory detachment affecting the OCTA flow signal [12]. Our results showed that the CCD of the fellow eyes and Sattler + Haller density did not decrease significantly during the follow-up period. We speculated that spironolactone could reduce the high permeability of choroidal vessels without causing damage or focal atrophy. In addition, short-term drug effects may not be able to completely reverse chronic choroidal vasodilation and high-density states. Long-term oral spironolactone may be necessary to reduce the hydrostatic pressure of the choroidal vessels. In addition, OCTA applies high-resolution volumetric flow information for motion contrast imaging [35]. Synchronous changes in choroidal vessel hyperpermeability and vasodilation may not change volumetric blood flow. Notably, oral administration of MR antagonists may be effective at promoting choriocapillaris recovery and reperfusion in the unaffected eyes of patients with chronic CSC [23].

In particular, the mean age of the patients in our study was 46 years, which was usually younger; however, in most similar studies, the mean age was 50 years. We have carefully reviewed this issue and the main reason for this is that the eight patients we included were younger than 40 years of age, and the youngest man was only 30 years old. All of these patients had a variety of risk factors, such as high work pressure, staying up late, and smoking. Similarly, other studies have reported a younger age of male CSC patients, such as 155 male CSC patients with a mean age of 43.8 ± 10.3 years and 75 male CSC patients with a mean age of 45.7 ± 8.9 years [36, 37]. Furthermore, 15 patients we included were over 50 years of age, but the oldest age was only 56 years old. This was due to the strict exclusion of any patient with signs of CNV or PCV in our patient screening. This also led to a younger age of the included patients, especially female patients, which was associated with a greater incidence of CNV (38, 39). In addition, another noteworthy point was the smaller male/female ratio (2.2/1) in our study population. The reported male-to-female ratio ranges from 2 : 1 to 6 : 1 according to previous studies [22]. A recent study reported a similar male/female ratio of 2.3/1 (63/27) compared with our study [38]. The main reason was that after informing the patient that there could be adverse endocrine system events after spironolactone administration, such as feminization of male breasts and erectile dysfunction, some young male CSC patients refused to take it. Elevated androgen levels (e.g., testosterone) were once thought to be associated with the pathogenesis of CSC. However, Subhi et al. explored the risk of CSC in a large cohort of male androgen abusers in a recent study. These authors speculated that androgen plays a subtle role at best in the pathophysiology of CSC [39]. Thus, we speculated that the potentially different effects on males and females caused by the weak estrogen-like effects of spironolactone might be relatively weak and had little function in therapy.

We must admit that there are inherent limitations of this study, including the small number of patients enrolled, the relatively short treatment time, and the absence of a placebo-treated group; thus, the recurrence rate and long-term efficacy could not be assessed. However, patients with a chronic course of CSC who cannot self-resolve were strictly selected for our study to avoid the influence of the natural history of CSC as much as possible. Furthermore, there was no exact treatment dose or regimen. All patients were treated by an experienced physician in a real-world practice environment, carefully ensuring patient's safety and rights. Third, the weak estrogen-like effects of spironolactone may have different effects on males and females, but the influence of spironolactone on therapeutic efficacy requires further exploration. Another limitation is the absolute error in manually demarcating the area of the SRF and the choroidal thickness, although segmentation software was used to repeat the measurements to ensure accuracy. Due to the lack of standardized CD analysis methods, vessel density data should be interpreted with caution, and these results cannot be compared with the results of other studies using different thresholds and calculation models [40, 41].

In conclusion, we report the good effect of oral spironolactone on the regression of SRF and the recovery of the anatomical structure of the retina in patients with chronic CSC. The baseline choroidal thickness and continuous ellipsoid zone seem to be useful imaging predictors for identifying chronic CSC patients who could have a potentially good response to spironolactone therapy.

## Figures and Tables

**Table 1:** Visual acuity and imaging data during the follow-up of patients with chronic CSC treated with spironolactone.

	Baseline	1 month	3 months	6 months
Complete resolution of SRF, *n* (%)		4 (9.5%)	20 (47.6%)	24 (57.1%%)

BCVA (logMAR) (decimal equivalent)	0.22 ± 0.27	0.21 ± 0.27	0.13 ± 0.30^†^	0.13 ± 0.25
	0.6 ± 0.5	0.6 ± 0.5	0.7 ± 0.5	0.7 ± 0.6

SRF area (mm^2^)	0.42 ± 0.25	0.20 ± 0.15^†^	0.12 ± 0.17^†^	0.07 ± 0.10^†^

CMT (*μ*m)	379.31 ± 98.40	290.77 ± 72.45	247.77 ± 88.21	223.03 ± 57.92^*∗*^

SFCT (*μ*m)	439.75 ± 125.63	368.13 ± 121.89	343.97 ± 104.24	349.09 ± 104.21^*∗*^

CCD (%) of fellow eyes	7.16 ± 3.88	7.01 ± 2.41	6.82 ± 1.64	6.88 ± 2.45

Sattler + Haller density (%)	5.83 ± 3.12	4.94 ± 2.18	4.96 ± 2.15	4.93 ± 1.88

CSC, central serous chorioretinopathy; SRF, subretinal fluid; BCVA, best-corrected visual acuity; CMT, central macular thickness; SFCT, subfoveal choroidal thickness; CCD, choriocapillaris density. ^†^*P* < 0.05 compared to baseline using the Wilcoxon paired signed-rank test. ^*∗*^*P* < 0.05 during the whole follow-up period according to repeated measure analysis of variance.

**Table 2:** Changes in visual acuity and imaging data between the responder group and the nonresponder group.

	Baseline	1 month	3 months	6 months
*Responder group, 24 eyes*				
BCVA (logMAR) (decimal equivalent)	0.17 ± 0.28	0.16 ± 0.28	0.09 ± 0.37	0.09 ± 0.21
	0.7 ± 0.5	0.7 ± 0.5	0.8 ± 0.4	0.8 ± 0.6
SRF area (mm^2^)	0.30 ± 0.14	0.13 ± 0.11	0.03 ± 0.05	0
CMT (*μ*m)	339.22 ± 77.75	261.50 ± 56.45	209.00 ± 40.18	189.44 ± 31.42
SFCT (*μ*m)	497.50 ± 99.36	388.50 ± 128.81	362.28 ± 102.02	364.67 ± 102.21
*Nonresponder group, 18 eyes*				
BCVA (logMAR) (decimal equivalent)	0.27 ± 0.27	0.26 ± 0.26	0.17 ± 0.16	0.18 ± 0.30
	0.5 ± 0.5	0.5 ± 0.5	0.7 ± 0.7	0.7 ± 0.5
SRF area (mm^2^)	0.58 ± 0.26^†^	0.28 ± 0.16^†^	0.24 ± 0.21^†^	0.15 ± 0.10^†^
CMT (*μ*m)	430.86 ± 100.49^*∗*^	328.39 ± 75.09^*∗*^	297.61 ± 108.10^*∗*^	266.21 ± 56.03^*∗*^
SFCT (*μ*m)	365.50 ± 119.04^*∗*^	341.93 ± 111.45	320.43 ± 106.01	329.07 ± 107.09

BCVA, best-corrected visual acuity; SRF, subretinal fluid; CMT, central macular thickness; SFCT, subfoveal choroidal thickness. ^†^*P* < 0.05 compared to the responder group using the Wilcoxon signed-rank sum test. ^*∗*^*P* < 0.05 compared to the responder group using an independent sample Student's *t*-test.

**Table 3:** Comparison of clinical characteristics between the responder group and the nonresponder group with chronic CSC treated with spironolactone.

	Responder group, 24 eyes	Nonresponder group, 18 eyes	*P* value
*Demographics*			
Age (y)	45.67 ± 5.37	46.57 ± 8.23	0.71^a^
Male (*n*)	16	13	>0.99^b^
Hypertension (*n*)	8	7	>0.99^b^
Disease duration (months)	7.22 ± 3.23	7.07 ± 2.73	0.89^a^
Baseline BCVA (logMAR)	0.17 ± 0.28	0.27 ± 0.27	0.13^c^
*Clinical characteristics*			
PED (*n*)	11	8	>0.99^b^
Double layer sign (*n*)	7	9	0.28^b^
Ellipsoid integrity	19	3	0.001^b^
ELM integrity	16	12	>0.99^b^
Hyperreflective foci in the OS and ON layers	17	15	0.43^b^
Subretinal deposits	8	10	0.28^b^
Baseline SFCT	497.50 ± 99.36	365.50 ± 119.04	0.002^a^
Baseline CCD (%)	6.71 ± 4.23	6.33 ± 3.83	0.80^a^
Baseline Sattler + Haller density (%)	6.21 ± 3.56	5.36 ± 2.47	0.64^c^
Abnormal choroidal vessels of choriocapillaris in OCTA	9	8	>0.99^b^

CSC, central serous chorioretinopathy; BCVA, best-corrected visual acuity; PED, pigment epithelial detachment; ELM, external limiting membrane; OS, outer segment; ON layer, outer nuclear layer; DD, disc diameter; SFCT, subfoveal choroidal thickness; CCD, choriocapillaris density; OCTA, optical coherence tomography angiography. ^a^Independent sample Student's *t*-test. ^b^Fisher's exact test. ^c^Wilcoxon signed-rank sum test.

## Data Availability

All data included in the statistical analysis are available upon request from the corresponding author.

## Abbreviations

AMD: Age-related macular degeneration
Anti-VEGF: Antivascular endothelial growth factor
BCVA: Best-corrected visual acuity
CMT: Central macular thickness
CNV: Choroidal neovascularization
CSC: Central serous chorioretinopathy
CCD: Choriocapillaris density
ELM: External limiting membrane
FFA: Fundus fluorescein angiography
ICGA: Indocyanine green angiography
MRs: Mineralocorticoid receptors
ON layer: Outer nuclear layer
OS: Outer segment
OCT: Optical coherence tomography
PCV: Polypoidal choroidal vasculopathy
PDT: Previous photodynamic therapy
PEDs: Pigment epithelium detachments
RPE: Retinal pigment epithelium
SFCT: Subfoveal choroidal thickness
SRF: Subretinal fluid.
